# Gangrène de la main après injection accidentelle intra artérielle de floxacilline: à propos d’un cas

**DOI:** 10.11604/pamj.2016.25.221.10803

**Published:** 2016-12-06

**Authors:** Tarik Salama, El Mohtadi Aghoutane, Rédouane El Fezzazi

**Affiliations:** 1 Service de Chirurgie Pédiatrique A, Départment des maladies de L’Enfant, CHU Mohammed VI, Université Cadi Ayyad, Marrakech, Maroc

**Keywords:** Injection intra artérielle, Floxacilline gangrène, main, Intra-arterial injection, Floxacilline, gangrene, hand

## Abstract

La floxacilline appartient à la classe des pénicillines bêta lactames. Dans notre contexte elle est très utilisée pour lutter contre les infections à germes Gram positif dont le staphylocoque doré. Cependant son utilisation doit être très prudente car elle n’est pas dénuée de complications. Nous rapportons l’observation d’un garçon de 6 ans opéré pour fracture de l’humérus. L’enfant a été mis sous floxacilline injectable après suspicion d’une infection sur matériel 2 mois après son opération. Le lendemain du début de l’antibiothérapie, l’enfant a présenté une ischémie aigue de la main droite. Il nous a alors été adressé pour prise en charge. Les explorations ont objectivé une obstruction de l’artère radiale. Une aponévrotomie de décharge a été réalisée et une héparinothérapie post opératoire a été démarrée. L’évolution a été marquée par une gangrène de toute la main. A travers cette observation nous voulons sensibiliser le personnel soignant sur le risque de survenue de cette complication désastreuse, et les mesures à prendre pour la prévenir.

## Abstract (en)

Floxacilline is a beta-lactam antibiotic of the penicillin class. In our context it is used to fight against infections caused by gram-positive bacteria, including staphylococcus aureus. However, floxacilline should be administered with great caution due to its possible side-effects. Our study reports the case of a 6-year old boy who underwent surgery for the treatment of a humeral fracture. The child was treated with injectable floxacilline following a suspected orthopaedic device infection 2 months after surgery. The day after starting antibiotic treatment, the child presented with acute ischemia of the right hand. Then, he was referred to us. Explorations objectified occlusion of the radial artery. The patient underwent relieving fasciotomy and received postoperative heparin. The evolution was marked by gangrene affecting the whole hand. The aim of our study was to educate caregivers about the risk of occurrence of this disastrous complication and about the measures to prevent it.

## Introduction

L’injection accidentelle intra-artérielle de substances est une complication rare mais bien réelle. Elle peut avoir des conséquences dramatiques. De nombreux cas ont été décrits suite à l’injection de drogues chez des toxicomanes. Des accidents peuvent également survenir dans le milieu hospitalier. Les premiers cas ont été décrits dans les années 1940. Les médicaments le plus fréquemment incriminés étaient les benzodiazépines et les barbituriques [1]. La floxacilline a été elle aussi mise en cause dans de nombreuses observations [2, 3]. Nous rapportons un nouveau cas de gangrène de la main, après injection intra-artérielle de Floxacilline, chez un garçon de 6 ans pris en charge dans notre structure. Notre but étant de sensibiliser encore plus le personnel soignant à cette complication dramatique.

## Patient et observation

**Figure 1:**
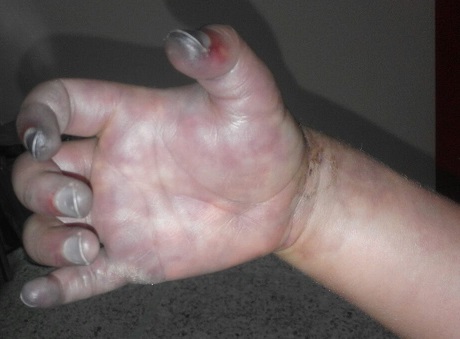
Main droite tuméfiée avec extrémités cyanosées

**Figure 2:**
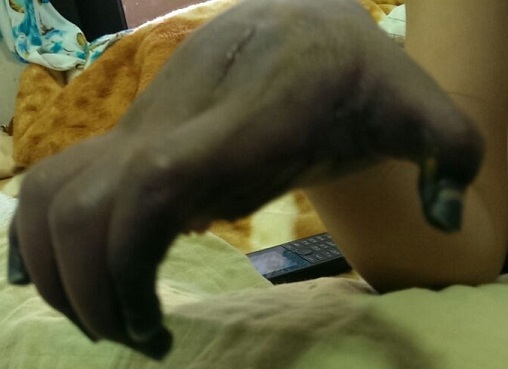
Nécrose étendue à toute la main

N.A, garçon de 6 ans, sans antécédents pathologiques notables, avait été hospitalisé dans notre service pour fracture de la diaphyse humérale, pour laquelle il a bénéficié d’une ostéosynthèse. Après 2 mois, l’évolution a été marquée par une infection de la plaie opératoire. Il a consulté dans un hôpital provincial proche de son domicile, où le diagnostic d’infection sur matériel a été retenu. Il a été hospitalisé et mis sous antibiothérapie par voie intra veineuse à base de floxacilline. Au 7^ème^ jour d’hospitalisation, alors qu’une nouvelle voie veineuse a été prise au niveau du poignet, du côté radial, l’enfant a présenté des douleurs intenses avec une cyanose des extrémités de la main droite. La douleur s’est intensifiée et la cyanose s’est étendue à toute la main dans les 2 jours suivants, avec froideur et hypoesthésie. Devant cette aggravation clinique le patient nous a été adressé pour prise en charge. A son admission dans notre structure l’enfant présentait une tuméfaction et une cyanose de toute la main droite, avec une nécrose des doigts (Figure 1). Le pouls ulnaire était bien perçu, le pouls radial était absent. L’écho doppler a objectivé une mauvaise individualisation de l’artère radiale avec absence de flux. Le patient a été acheminé d’urgence au bloc opératoire où une aponévrotomie de décharge a été réalisé; une héparinothérapie a été démarrée en post opératoire immédiat à raison de 150 UI/kg/jour. Les jours suivants ont été marqués par l’extension de la nécrose à toute la main (Figure 2). Nous nous sommes résignés à une amputation au niveau du poignet.

## Discussion

De nombreux cas de complications vasculaires des membres, suite à l’injection de substances en intra artériel, ont été décrits dans la littérature. Il s’agit le plus souvent d’accidents de prises de drogues lors de conduites addictives [4–6]. Certains médicaments ont également été incriminés, tel que les corticostéroïdes [7], la prométhazine [8] et la floxacilline [3]. Plusieurs hypothèses ont été proposées pour expliquer la survenue de ces complications. Une mauvaise dilution de la préparation pourrait favoriser une agrégation des molécules ou une cristallisation de la substance [9], entrainant ainsi une thrombose vasculaire. Une lésion directe de la paroi vasculaire ou des modifications anatomiques de la vascularisation pourrait favoriser la thrombose artérielle [2, 5]. Un vasospasme réflexe pourrait également être à l’origine de cette ischémie [10]. Sur un plan clinique, le maître symptôme est la douleur aigue, lancinante. Elle sera suivie d’une cyanose rapidement extensive des extrémités vers la racine du membre. Apparaissent également une tuméfaction et des troubles de la sensibilité. Aucun consensus n’est actuellement établit pour prendre en charge cette ischémie aigue. Le principe de tout traitement étant de préserver le flux sanguin distal et de prévenir le vasospasme [4]. Pour ce faire, une surélévation du membre est réalisée; l’administration d’héparine ou de corticostéroides peut également être tentée. L’utilisation de blocs nerveux pour lutter contre le vasospasme, et contre la douleur [4] peut s’avérer utile. D’autres traitements ont été utilisés avec succès; on citera entre autres les Prostacycline analogues, les vasodilatateurs artériels, les inhibiteurs de thromboxane et l’oxygénothérapie hyperbare [2, 5]. Dans un article publié à propos d’un cas d’ischémie aigue de la main après injection artérielle de poudre de Méprobamate, Seak et al ont proposé un algorithme de prise en charge de ces ischémies, regroupant la plupart des possibilités thérapeutiques à notre disposition [4] (Figure 3). Dans les cas extrêmes, l’aponévrotomie, voir l’amputation peut être nécessaire.

## Conclusion

L’administration de substances en intra artériel peut avoir des conséquences dramatiques. Bien que de nombreux traitements soient proposés, aucun consensus n’est établit. Le pronostic reste très réservé. Le meilleur traitement demeure la prévention. D’où la nécessité de lutter contre la banalisation de l’injection intra veineuse, et de sensibiliser le personnel soignant aux risques bien réels. De simples précautions peuvent éviter un drame; il suffit de s’assurer de la bonne dilution de la solution à injecter; et par une simple aspiration, prendre la peine de vérifier que notre aiguille est bien dans une veine.
